# Exploring the Association Between *CD36* rs1761667 Polymorphism and Susceptibility to Non-Contact Tissue Injuries in Moroccan Elite Cyclists and Field Hockey Players: A Pilot Study

**DOI:** 10.3390/genes16060651

**Published:** 2025-05-28

**Authors:** El Mokhtar El Ouali, Jihan Kartibou, Juan Del Coso, Badreddine El Makhzen, Laila Bouguenouch, Ramzi El Akbir, Abdelmoujoud El Haboussi, Omar Akhouayri, Azeddine Ibrahimi, Abdelhalem Mesfioui, Hassane Zouhal

**Affiliations:** 1 Sports Science Research Team, Institute of Sports Sciences, Hassan I University, Settat 26002, Morocco; abdelmoujoud.elhaboussi@uhp.ac.ma; 2 Department of Biology, Laboratory of Biology and Health, Ibn Tofail University of Kenitra, Kenitra 14000, Morocco; 3 Sport Sciences Research Centre, Rey Juan Carlos University, 28942 Fuenlabrada, Spain; juan.delcoso@urjc.es; 4 Medical Genetics Unit, Central Laboratory, CHU Hassan II, Faculty of Medicine, Pharmacy and Dentistry, Sidi Mohamed Ben Abdellah University, Fez 30000, Moroccolaila.bouguenouch@usmba.ac.ma (L.B.);; 5 Medical Biotechnology Laboratory, Faculty of Medicine and Pharmacy, Mohamed 5 Rabat University, Rabat 10000, Morocco; 6 University Rennes, M2S (Laboratoire Mouvement, Sport, Santé)—EA 1274, 35000 Rennes, France; hassane.zouhal@univ-rennes2.fr; 7 Institut International des Sciences du Sport (2I2S), 35850 Irodouer, France

**Keywords:** cluster of differentiation 36, endurance athletes, athletic performance, team sports athletes, tissue damage

## Abstract

**Background**: Non-contact tissue injury in elite athletes is influenced by multiple factors, including genetic predisposition. Although previous research has identified several genetic markers associated with injury susceptibility, the role of the *CD36* (cluster of differentiation 36) gene, a key regulator of fatty acid transport into skeletal muscle and other vital tissues, remains unexplored in this context. A single-nucleotide polymorphism in the *CD36* gene (rs1761667) involves an A-to-G substitution (with three genotypes = AA and GG homozygotes and AG heterozygotes), and previous data have reported that individuals carrying the AA genotype of the *CD36* gene show reduced expression of the CD36 protein and poorer lipid metabolism. Additionally, it has been recently found that the frequency of the AA genotype is significantly lower in elite cyclists compared to field hockey players. No previous study has examined the association between the *CD36* rs1761667 polymorphism and athlete injury risk. Therefore, the aim of this study was to investigate the potential association between the *CD36* rs1761667 polymorphism and non-contact tissue injury susceptibility in elite Moroccan cyclists and field hockey players. **Methods**: Forty-three elite Moroccan male athletes, including 19 cyclists and 24 national team field hockey players, volunteered for this study. Non-contact tissue injuries during the 2022/2023 sports season have been recorded. Genotyping of the *CD36* rs1761667 polymorphism was carried out using Sanger sequencing. Chi-square tests were used to analyze the Hardy–Weinberg equilibrium and compare the genotypes and characteristics of athletes with and without non-contact injuries. **Results**: During the 2022/2023 sports season, 21.05% of cyclists (4 out of 19) and 33.33% of field hockey players (8 out of 24) experienced non-contact tissue injuries. The genotypic frequency was similar in the injured and non-injured groups among cyclists (χ^2^ and *p* not calculated because “AA = 0” in both groups), field hockey players (χ^2^ = 3.30, *p* = 0.19), and all athletes (χ^2^ = 1.73, *p* = 0.41). Additionally, the dominant model of the *CD36* rs1761667 polymorphism (AA+AG vs. GG) did not reveal a significant risk of non-contact injuries among cyclists (OR: 1.20, 95% CI: 0.13–19.09, *p* > 0.9999), field hockey players (OR: infinity, 95% CI: 0.23-infinity, *p* = 0.53), and all athletes (OR: 2.75, 95% CI: 0.32–34.12, *p* = 0.65). Furthermore, the recessive model (AA vs. AG+GG) did not demonstrate any effect on the risk of non-contact injuries in cyclists (OR and 95% CI not calculated, *p* > 0.9999), field hockey players (OR: 0.33, 95% CI: 0.05–2.40, *p* = 0.38), and all athletes (OR: 0.55, 95% CI: 0.10–2.60, *p* = 0.69). **Conclusions**: This study suggests that the association between specific genotypes (AA, AG, and GG) or alleles (A and G) of the *CD36* gene and susceptibility to non-contact tissue injuries in Moroccan cycling and field hockey players is uncertain. Given the small sample size, further studies will be needed to explore and confirm these findings.

## 1. Introduction

Professional sport is characterized by a very intense and high level of competition, where athletes frequently push their physical and physiological limits to achieve optimal performance. Maintaining and improving these extraordinary levels of performance requires the implementation of comprehensive and carefully designed training programs. The accumulation of physical and physiological stress, combined with insufficient recovery periods after training or competition, can promote the development of significant non-contact tissue injuries.

Non-contact injuries are characterized by indirect tissue damage occurring without direct external trauma [1]. In addition, non-contact injuries are generally due to a systemic failure resulting from over-exertion during exercise, such as structural tissue damage [2,3]. Non-contact injuries typically encompass a wide range of body tissues, including ligaments [4], bones [5], tendons [6], nerves [7], and muscles [8]. Among these, muscle injuries stand out as the most common type of non-contact injuries in elite athletes. Overall, around 92% of indirect muscle injuries mainly affect the four main muscle groups of the lower limbs. Of these, the hamstrings are the most frequently affected (37%), followed by the adductors (23%), quadriceps (19%), and calf muscles (13%) [1]. Interestingly, various factors can affect the appearance and severity of non-contact tissue injuries in elite athletes, including performance capacity, training method, recovery, nutrition, and individual intrinsic variations [9,10,11]. Furthermore, according to previous studies, genetic factors may also play an important role in the occurrence and severity of non-contact tissue injuries [10,12,13].

Several genetic polymorphisms have been found to correlate with increased susceptibility to non-contact injuries in elite athletes [14,15,16,17]. Among these, some are associated with the gene-encoding proteins involved in substrate transport, metabolism, and energy provision for working muscles. The *CD36* gene encodes a transmembrane glycoprotein called cluster of differentiation 36 (CD36), also known by several other names, including fatty acid translocase (FAT), FAT/CD36, and platelet glycoprotein IV [18]. The *CD36* gene is located on the long arm of chromosome 7, at band q11.21 (7q11.21) [19]. This gene consists of 15 exons coding for a single chain comprising 472 amino acids [20,21]. The CD36 protein is present in a variety of tissues, including blood cells, adipose tissue, the endothelium, the liver, the heart, and skeletal muscle [22,23]. Interestingly, the CD36 protein is the major transporter of long-chain fatty acids (LCFAs), mediating their transport from adipose tissue to vital sites, as well as to skeletal muscle cells and mitochondria [24,25,26]. During endurance exercise, the CD36 protein has been demonstrated to play a critical role in the lipolysis process in adipose tissue and the transport of fatty acids to the mitochondria for oxidation [27,28,29]. In addition, a correlation was observed between increased fat oxidation and higher levels of CD36 protein during physical effort [30]. Furthermore, an increase in CD36 protein levels has been reported in human skeletal muscle after endurance cycling exercise carried out at 60% of peak oxygen uptake (VO_2_ peak) [31]. These findings highlight the importance of the CD36 protein in the regulation of energy metabolism and its potential to impact the athletic performance of athletes.

A single nucleotide polymorphism (SNP) in the *CD36* gene, known as rs1761667, involves an A-to-G substitution [32]. This polymorphism gives rise to three possible genotypes: AA and GG homozygotes and AG heterozygotes [32]. Generally, individuals carrying the AA genotype of the *CD36* gene show reduced expression of the CD36 protein, as well as its activity [33]. Furthermore, individuals with deficient CD36 protein activity may have deleterious changes in lipid metabolism [34], which increases the risk of suffering from various pathologies [35,36]. From a physiological perspective, the AA genotype may be associated with reduced exercise performance, as a lower expression of the CD36 protein can impair the transport of fatty acids to the mitochondria for oxidation, particularly when compared to individuals with the GG or GA genotypes. This effect may be more pronounced in endurance-based sports, where energy provision for aerobic exercise relies more heavily on fat oxidation than in high-intensity intermittent activities such as team sports. Interestingly, it has been recently found that the frequency of the AA genotype of the *CD36* gene is significantly lower in elite cyclists compared to field hockey players [37], supporting this theory. The AA genotype of the *CD36* gene may be associated with other negative phenotypes, such as increased susceptibility to injury. This may be due to less-efficient fat oxidation during exercise, which can lead to earlier reliance on glycogen stores, accelerated fatigue, and reduced energy availability, particularly during prolonged aerobic activity. However, to the authors’ knowledge, no previous study has examined the association between the *CD36* rs1761667 polymorphism and injury risk in athletes. Consequently, the aim of this study was to explore the potential association between the *CD36* rs1761667 polymorphism and susceptibility to non-contact tissue injuries in Moroccan elite athletes, particularly for cyclists and field hockey players. Based on the physiological effects of the *CD36* rs1761667 polymorphism mentioned above, we hypothesized that the AA genotype would be associated with greater susceptibility to non-contact injuries, due to reduced *CD36* expression and impaired fatty acid oxidation. This association was expected to be observed only in cyclists, given the greater reliance on fat as an energy source in endurance-based cycling compared to field hockey.

## 2. Materials and Methods

### 2.1. Ethics Statement

The study received approval from the Research Ethics Committee of the Ibn Tofail University Doctoral Center and adhered to the established guidelines for biomedical research involving human subjects (approval number: 23/2020, date of approval: 24 September 2020). Detailed explanations of the procedures, as well as the potential risks and benefits associated with participation, were carefully communicated to all participants. Before participating, the participants voluntarily gave informed consent by signing a consent form, signifying their willingness to participate in the study. The procedures followed the International Federation of Sports Medicine consensus statement on genetic information [38] and were conducted in accordance with the principles outlined in the Declaration of Helsinki (1975, revised in 2013). As a reward for participating in the study, all athletes received a personalized report containing information on the distribution of their *CD36* gene. This was given to them after the conclusion of the study.

### 2.2. Study Design and Participants

Forty-three Moroccan elite male athletes participated voluntarily in this study. No a priori power calculation was performed due to the exploratory nature of the study and the absence of prior effect size estimates in this specific population. A convenience sampling method was used, based on the availability of eligible participants during the recruitment period. Among the sample of participants, 19 were cyclists, selected from the Moroccan national cycling team. Notably, two of these cyclists had successfully secured qualification for the Paris 2024 Olympic Games, while six had qualified for the 2023 World Championships, and two had previously emerged victorious in the Tours of Africa. The remaining 24 athletes were members of the Moroccan national field hockey team. According to the most recent continental and international rankings, the field hockey team held the sixth position in Africa and the forty-ninth position worldwide. All participants underwent anthropometric measurements in a standardized manner, ensuring uniform conditions for all participants. Trained nurses took 4 mL blood samples from each participant, following strict safety protocols. To ensure confidentiality, an alphanumeric code was assigned to each blood sample. The tubes containing the samples were then stored at −80 °C to preserve their integrity for later analysis.

### 2.3. Injury Collection

Information regarding non-contact injuries was collected prospectively from all athletes. In collaboration with the medical staff of the Moroccan national cycling and field hockey federations, diagnosed sports-related injuries were classified and documented using the classification system established by the medical commission of the International Olympic Committee (IOC), including a cycling-specific extension [39]. According to the IOC consensus statement, a non-contact injury is defined as an injury that occurs in the absence of direct contact with another athlete, object, or surface, typically resulting from intrinsic factors such as neuromuscular control, biomechanical patterns, or fatigue [40]. During the 2022/2023 sports season, which ran from September 2022 to July 2023 for cycling and from August 2022 to June 2023 for field hockey, non-contact injuries were carefully documented in all athletes. The injury recording specifically focused on non-contact injuries sustained during training or competition as part of sports exposure, which were diagnosed by qualified medical professionals. Injuries resulting from collisions with other cyclists/players or with objects, directly or indirectly, were deliberately excluded from the survey, as they were deemed less likely to be influenced by the athlete’s genotype. To facilitate consistent data collection, the teams’ medical staff used a custom injury-reporting questionnaire developed by the research team. This tool was completed shortly after each non-contact injury incident throughout the season. For the purposes of this investigation, the sample population was classified into two distinct groups: (a) the injured group, comprising all athletes who suffered at least one non-contact tissue injury during the season, and (b) the non-injured group, comprising athletes who had not reported any non-contact tissue injuries during the sports season.

### 2.4. Genotyping

Genomic DNA extraction was performed on leukocyte samples using a commercial kit, specifically the MagPurix Blood DNA Extraction Kit (Brussels, Belgium), according to the manufacturer’s guidelines. Subsequently, the DNA concentrations were assessed using a microspectrophotometer via a nanodrop assay. Genotyping procedures for the *CD36* SNP rs1761667 (G > A) were performed by polymerase chain reaction (PCR) using a thermal cycler, which included the VERITYTM instrument manufactured by Applied Biosystems (Waltham, MA USA). The primers used in this study have previously been described [41]: forward primer: 5′-CAAAATCACAATCTATTCAAGACCA-3′ and reverse primer: 5′-TTTTGGGAGAAATTCTGAAGAG-3′. The PCR process commenced with an initial heating phase at 94 °C, maintained for 5 min to denature the DNA strands. Following this, a series of 30 amplification cycles was conducted. Each cycle involved a brief denaturation step at 94 °C for 30 s to separate the DNA strands, followed by an annealing phase at 55 °C for 30 s where the primers bind to their complementary sequences on the DNA template, and subsequently, an extension phase at 72 °C for 30 s to allow the DNA polymerase to extend the DNA strands. Lastly, a final extension step was performed at 72 °C for 4 min to ensure all remaining single-stranded DNA is fully extended. Furthermore, after PCR amplification, the products obtained were subjected to analysis on a 2% agarose gel, in which a size marker was included to serve as a reference to determine the molecular weight. Electrophoresis was used to separate the DNA fragments according to their size. The separated fragments were visualized under UV light, facilitated by the intercalation of ethidium bromide. This process was used to evaluate the quality, quantity, and size distribution of the amplicons. Once the PCR procedure was completed, enzymatic purification was performed using ExoSAP-IT™ (Waltham, MA USA) to remove and neutralize any residual PCR components. Subsequently, the amplified DNA underwent sequencing using the BigDye^®^ Terminator v. Cycle Sequencing Kit 3.1 (Waltham, MA USA), manufactured by Applied Biosystems. Finally, the acquired sequences were analyzed using Sequencing Analysis software, version 3.4.

### 2.5. Statistical Analysis

The conformity of the samples with the genotypic frequencies of the Hardy–Weinberg equilibrium (HWE) was assessed using a chi-square (χ^2^) test. This assessment consisted of comparing the observed genotype frequencies within each group with the expected genotype frequencies derived from the principles of the Hardy–Weinberg equilibrium. To analyze the categorical variables and compare the genotypic and allelic frequencies across all groups, the χ^2^ test was used. Additionally, to compare the genotypes (AA, AG, and GG) and alleles (A and G) between the injured and non-injured groups within the cycling, field hockey, and all athletes’ groups, χ^2^ was also used. To evaluate dominant (AA+AG vs. GG) and recessive (AA vs. AG+GG) models within the injured and non-injured groups, Fisher’s exact test was applied, and odds ratios (OR) with intervals of 95% confidence (CI) were calculated to verify the influence of these models on susceptibility to non-contact injuries. Continuous variables, such as anthropometric data, were assessed for normality by visual inspection (QQ-Plots) and the D’Agostino–Pearson test. When comparing groups based on anthropometric data, a one-way analysis of variance (ANOVA) was used for normally distributed data, while the Kruskal–Wallis test was used for the data that did not adhere to a normal distribution. The results are presented as means and standard deviations (SD) for normally distributed data and as medians and interquartile ranges (IQR, Q1–Q3) for non-normally distributed data. The statistical significance threshold was set at *p* < 0.05 for all analyses. Statistical analysis was performed using GraphPad Prism 9.2.0 (GraphPad Software Inc., San Diego, CA, USA).

## 3. Results

From the total sample of 43 participating athletes, 12 athletes suffered a non-contact injury during the 2022/2023 sports season (injured group), including 4 cyclists (representing 21.05% of the cyclists’ sample) and 8 field hockey players (representing 33.33% of the field hockey players’ sample). Conversely, the remaining 31 athletes formed the non-injured group, comprising 15 cyclists and 16 field hockey players. Regarding the anthropometric measurements, no significant differences were observed between the injured and non-injured groups (*p* > 0.05; Table 1).

**Figure 1:**
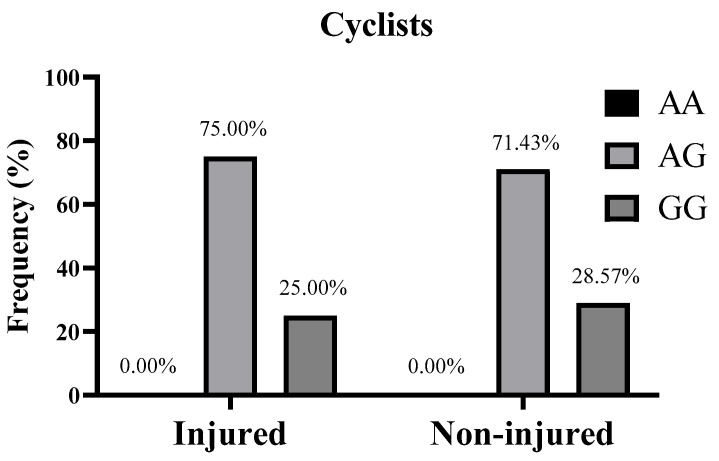
Genotypic frequency of *CD36* rs1761667 polymorphism in cyclists (n = 18) with and without non-contact injuries during the 2022–2023 sports season. Genotype distribution of injured vs. non-injured athletes was compared with a Chi-square test (χ^2^).

**Figure 2:**
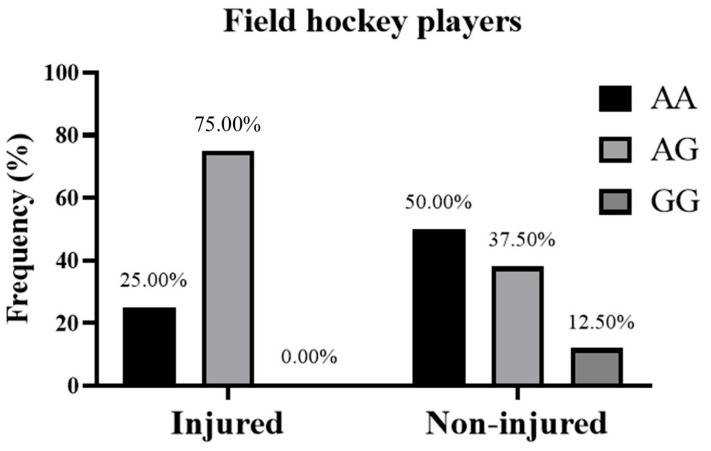
Genotypic frequency of *CD36* rs1761667 polymorphism in field hockey players (n = 24) with and without non-contact injuries during the 2022–2023 sports season. Genotype distribution of injured vs. non-injured athletes was compared with a Chi-square test (χ^2^).

**Figure 3:**
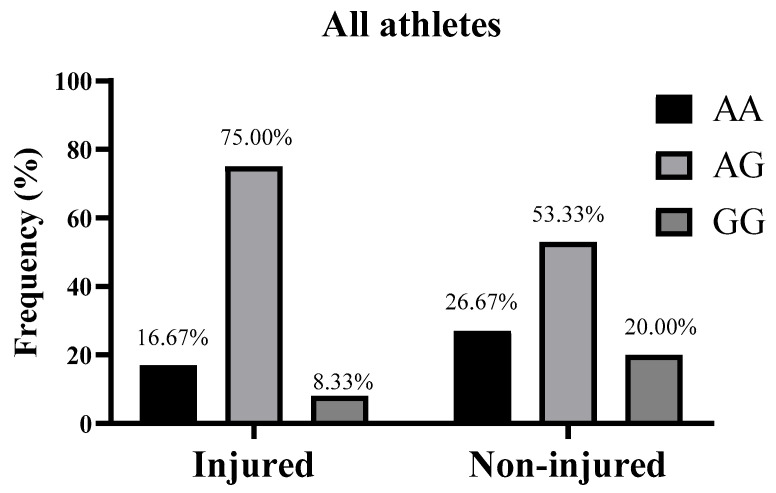
Genotypic frequency of *CD36* rs1761667 polymorphism in all athletes (n = 42) with and without non-contact injuries during the 2022–2023 sports season. Genotype distribution of injured vs. non-injured athletes was compared with a Chi-square test (χ^2^).

Genotyping of the *CD36* gene was successfully performed for all participants, except for one DNA sample in the cycling group, which belonged to a cyclist who did not report any injury. The distribution of the *CD36* rs1761667 polymorphism in all athletes was not significantly deviated from the HWE (*p* = 0.44). Moreover, the genotypic frequency of *CD36* rs1761667 polymorphism in the injured and non-injured groups was as follows. For cyclists, the *CD36* genotype distribution was described as AA/AG/GG, 0.00/75.00/25.00% in the injured group and 0.00/71.43/28.57% in the non-injured group (Figure 1). For field hockey players, it was 25.00/75.0/0.00% in the injured group and 50.00/37.50/12.50% in the non-injured group (Figure 2). In the all athletes group, it was 16.67/75.00/8.33% for the injured group and 26.67/53.33/20.00% in the non-injured group (Figure 3). Furthermore, in terms of allelic frequency in the injured and non-injured groups, the following data were obtained: in the cyclists, the *CD36* alleles as A/G were 37.50/62.50% in the injured group and 35.71/64.29% in the non-injured group. Among the field hockey players, it was 62.50/37.50% in the injured group and 68.75/31.25% in the non-injured group, and for all athletes, it was 54.17/45.83% for the injured group and 53.33/46.67% in the non-injured group, respectively (Table 2). Generally, there were no significant differences in the *CD36* genotypes between the injured and non-injured groups for cyclists (χ^2^ and *p* not calculated because “AA = 0” in both groups), field hockey players (χ^2^ = 3.30, *p* = 0.19), and all athletes (χ^2^ = 1.73, *p* = 0.41). Moreover, the dominant model of the *CD36* rs1761667 polymorphism (AA+AG vs. GG) did not indicate a significant risk of non-contact injuries in cyclists (OR: 1.20, 95% CI: 0.13–19.09, *p* > 0.9999), field hockey players (OR: infinity, 95% CI: 0.23–infinity, *p* = 0.53) and the whole group of athletes (OR: 2.75, 95% CI: 0.32–34.12, *p* = 0.65). Similarly, the recessive model (AA vs. AG+GG) did not show a statistically significant association with non-contact injuries in cyclists (OR and 95% CI not calculated, *p* > 0.9999), field hockey players (OR: 0.33, 95% CI: 0.05–2.40, *p* = 0.38), and in all athletes (OR: 0.55, 95% CI: 0.10–2.60, *p* = 0.69), (Table 3).

## 4. Discussion

The purpose of this study was to explore the potential role of the *CD36* rs1761667 polymorphism in Moroccan elite athletes by comparing those who had experienced non-contact injuries with those who had not. Our findings indicate that the frequencies of genotypes (AA, AG, and GG) and alleles (A and G) of the *CD36* rs1761667 polymorphism were similar in both groups (injured and non-injured) of athletes. Interestingly, no significant differences in *CD36* genotype distribution were observed between the injured and non-injured athletes in either the cycling or field hockey groups. These two sports differ markedly in terms of exercise intensity, metabolic demands, energy provision, and training and competition profiles. Given the functional implications of the *CD36* rs1761667 polymorphism, we hypothesized that the AA genotype, which is associated with lower *CD36* expression and reduced transport of fatty acids to the mitochondria, might increase injury susceptibility in cyclists, who rely more heavily on fat oxidation during prolonged aerobic efforts. While the overall injury prevalence was higher in field hockey than in cycling (33.33% vs. 21.05%, respectively), the polymorphism did not influence the distribution of genotypes between the injured and non-injured athletes in either sport. Collectively, these results may suggest that the association between the *CD36* gene and susceptibility or protection against non-contact tissue injuries is uncertain. More specifically, given the pilot nature of this study, this suggestion relates specifically to elite Moroccan athletes engaged in the disciplines of cycling and field hockey. The generalization of these results to athletes of different origins and sports disciplines represents a major challenge that should be resolved with further investigation.

The *CD36* gene is located on the outer mitochondrial membrane and plays a crucial role in fatty acid activation, regulation, and transport [42]. In addition, an association was noted between elevated lipid oxidation and increased CD36 protein content during physical exercise [30]. However, this finding highlights the potential importance of the *CD36* gene in energy production during exercise and its implications for athletes’ sports performance. Furthermore, after endurance cycling exercise, an increase in the CD36 protein levels in the human skeletal muscle was observed [31]. Generally, the AA genotype of the *CD36* gene is associated with reduced CD36 protein expression and activity compared to the G allele [33]. Interestingly, a dominance of the G allele was observed in elite cyclists [37]. Conversely, the AA genotype and the A allele are over-represented in field hockey players [37]. These results are probably due to the greater demand for fatty acid oxidation in cycling compared with hockey, combined with the increased CD36 protein content and activity associated with this metabolic pathway. On the other hand, the current study represents the first investigation into the prevalence of genotypes and alleles of the *CD36* rs1761667 polymorphism in elite athletes and its potential association with susceptibility to non-contact injuries. Therefore, the ability to engage in full discussion and compare the current results with previous data is limited by the lack of studies on the *CD36* gene and its relationship to non-contact tissue injuries in elite athletes. Consequently, the main findings of this study will remain in the realm of hypotheses until they are confirmed or contradicted by other studies. Conversely, previous research on the *CD36* gene has often focused on studying patients in order to assess its potential association with specific diseases. For instance, an association between the *CD36* rs1761667 polymorphism and susceptibility to hypertension has been highlighted [43]. Furthermore, it has been noted that the AA genotype of *CD36* rs1761667 polymorphism exhibited a higher prevalence among the Moroccan obese group compared to the normal weight group [44]. Last, the AA genotype was found to be correlated with high levels of serum triglycerides and total cholesterol [32]. Given these studies, it is conceivable that the AA genotype of the *CD36* gene, known to be significantly associated with various pathologies, may also correlate with non-contact injuries in elite athletes. However, this was not observed in our athletic population, likely because regular exercise training may attenuate some of the deleterious metabolic effects associated with the AA genotype and the A allele of this gene. Moreover, while previous associations of this genotype have primarily involved systemic cardiovascular and metabolic diseases, our study focused on local musculoskeletal outcomes, which may be influenced by distinct mechanisms. It is still important to note that several factors could influence these results. These include the physical and physiological demands of different sporting disciplines, as well as the type, location, and severity of specific injuries. In addition, the ethnic origin of athletes, as well as other environmental variables, may also play a significant role in determining these outcomes. Given these complexities, further research is needed to confirm this hypothesis and better understand the potential link between the *CD36* gene and injury susceptibility in elite athletes.

### Limitations

Although this study is the first to investigate the relationship between the *CD36* rs1761667 polymorphism and susceptibility to non-contact tissue injuries in elite cyclists and field hockey players, it is not without limitations. First, the small sample size (n = 43) and the use of convenience sampling may have limited the statistical power of the study. This is evident in the wide confidence intervals observed around the odds ratios, which are indicative of low statistical power and increase the uncertainty in determining whether an association exists between the *CD36* rs1761667 polymorphism and the risk of non-contact injuries in elite athletes. Moreover, the lack of studies on the association of the *CD36* gene and the risk of non-contact injuries in elite athletes has limited the discussion and the potential applications of the study. Additionally, the analysis employed in this study did not take into account the volume and intensity of the training factors that could have contributed to a more complete understanding of the relationship between training load, the emergence of non-contact injuries, and a potential genetic predisposition. Furthermore, the current study exclusively targets male athletes, thus potentially limiting the generalizability of study results to female athletes, whose physiological and genetic profiles may differ. Finally, non-contact tissue injuries that occur during training and competition are a multifactorial event, and their origins may involve various genes and polymorphisms and environmental factors, and their complex interactions [45,46,47]. Consequently, focusing on a single polymorphism may be considered a limitation of this study.

## 5. Conclusions

In summary, the distribution of genotypes and alleles of the *CD36* rs1761667 polymorphism in athletes with and without non-contact tissue injuries was similar overall and for the sample of cyclists and field hockey players. Therefore, the association between specific genotypes (AA, AG, and GG) or alleles (A and G) of the *CD36* gene and susceptibility to non-contact injuries in Moroccan cycling and field hockey players has not been confirmed. As this is a pilot study with a relatively small sample size, the findings should be interpreted with caution. Further studies with larger, more diverse cohorts (including male and female athletes from various sports disciplines and ethnic backgrounds) are needed to validate these preliminary observations and clarify the potential role of the *CD36* polymorphism in injury susceptibility among elite athletes. Until further data are available, genotyping of the *CD36* gene does not appear to be clinically useful for talent identification or for predicting injury risk in athletes. Instead, it may be more valuable to explore genetic variants in other genes that have demonstrated stronger associations with performance traits or injury susceptibility, such as *ACTN3*, *COL5A1*, and *ACE*.

## Figures and Tables

**Table 1:** Age and anthropometric variables of Moroccan elite cyclists and field hockey players with and without non-contact injuries during the 2022–2023 sports season.

	All Athletes	Injured	Non-Injured	*p*-Value
**Age (yr)**	20 (18–23)	21.83 ± 3.86	20 (18–23)	0.80
**Weight (kg)**	64.30 ± 6.23	65.83 ± 4.59	64 (60–67)	0.22
**Height (m)**	1.77 ± 0.05	1.78 ± 0.04	1.76 ± 0.05	0.65
**BMI (kg/m^2^)**	20.64 ± 1.93	20.87 ± 1.43	20.55 ± 2.11	0.89

BMI: Body mass index.

**Table 2:** Genotypic and allelic frequencies of *CD36* rs1761667 polymorphism in Moroccan elite cyclists and field hockey players with and without non-contact injuries during the 2022–2023 sports season.

Groups			Cyclists	Field Hockey Players	All Athletes
			n = 18	n = 24	n = 42
**Injured**	**Genotype n (%)**	AA	0 (0.00%)	2 (25.00%)	2 (16.67%)
		AG	3 (75.00%)	6 (75.00%)	9 (75.00%)
		GG	1 (25.00%)	0 (0.00%)	1 (8.33%)
	**Allele n (%)**	A	3 (37.50%)	10 (62.50%)	13 (54.17%)
		G	5 (62.50%)	6 (37.50%)	11 (45.83%)
**Non-injured**	**Genotype n (%)**	AA	0 (0.00%)	8 (50.00%)	8 (26.67%)
		AG	10 (71.43%)	6 (37.50%)	16 (53.33%)
		GG	4 (28.57%)	2 (12.50%)	6 (20.00%)
	**Allele n (%)**	A	10 (35.71%)	22 (68.75%)	32 (53.33%)
		G	18 (64.29%)	10 (31.25%)	28 (46.67%)

The CD36 genotype could not be clearly determined in the DNA sample of one cyclist who did not report any injury. Therefore, this table includes data from only 42 athletes, of whom 18 are cyclists.

**Table 3:** The association between *CD36* rs1761667 polymorphism and non-contact injuries in elite Moroccan cyclists and field hockey players.

Groups	Injured vs. Non-Injured			Dominant (AA+AG vs. GG)		Recessive (AA vs. AG+GG)	
	χ^2^	df	*p*-Value	OR [95% CI]	*p*-Value	OR [95% CI]	*p*-Value
**Cyclists**	-	-	-	1.20 [0.13–19.09]	>0.9999	-	>0.9999
**Field hockey** **players**	3.3	2	0.19	infinity [0.23–infinity]	0.53	0.33 [0.05–2.40]	0.38
**All athletes**	1.73	2	0.41	2.75 [0.32–34.12]	0.65	0.55 [0.10–2.60]	0.69

df: degree of freedom. In cyclists, the *p*-value for the chi-square test was not calculated due to the absence of cyclists with the AA genotype in both injured and non-injured groups (frequency = 0; see Table 2 for further details).

## Data Availability

The raw data supporting the conclusions of this article will be made available by the authors upon request.

## Abbreviations

CD36	cluster of differentiation 36
FAT	fatty acid translocase
IOC	International Olympic Committee
LCFAs	long-chain fatty acids
PCR	polymerase chain reaction
SNP	Single-nucleotide polymorphism
VO_2_ peak	peak oxygen uptake
